# Distinct circulating monocyte profiles in chronic cannabis users compared to cocaine users without changes in plasma levels of proinflammatory cytokines and LPS

**DOI:** 10.1515/nipt-2025-0003

**Published:** 2025-04-02

**Authors:** Douglas Johnson, Zhenwu Luo, Sylvia Fitting, Zizhang Sheng, Wei Jiang

**Affiliations:** Department of Pharmacology and Immunology, 2345 Medical University of South Carolina, Charleston, USA; Ralph H. Johnson VA Medical Center, Charleston, USA; Department of Psychology & Neuroscience, University of North Carolina at Chapel Hill, Chapel Hill, NC, USA; Aaron Diamond AIDS Research Center, Columbia University Vagelos College of Physicians and Surgeons, New York, NY, USA

**Keywords:** cocaine, cannabis, monocyte, inflammation

## Abstract

**Objectives:**

Chronic cannabis use is linked to anti-inflammatory effects, and cocaine exhibits context-dependent immunomodulation; their distinct impacts on monocyte subsets and systemic inflammation are not fully understood. Systemic microbial translocation contributes to monocyte differentiation, but the levels in chronic cocaine and cannabis users in humans *in vivo* are lacking.

**Methods:**

Peripheral blood mononuclear cells (PBMCs) and plasma samples were collected from chronic cocaine users, cannabis users, and non-drug users. The route of drug administration was via smoking or snorting. Monocyte subsets were analyzed using flow cytometry; plasma levels of cytokines (IL-2, IL-4, IL-6, IL-10, TNF-α, and IFN-γ) and lipopolysaccharide (LPS, a marker of microbial translocation) were measured using a Meso Scale immunoassay and Limulus amoebocyte lysate assay, respectively.

**Results:**

Cannabis use was associated with increased total monocyte counts, increased percentages of a classical subset (CD14++CD16−), and decreased percentages of a non-classical subset (CD14+CD16++) in CD14+ monocytes compared to cocaine users and/or healthy controls. Similar levels were observed in the percentages of intermediate monocytes (CD14++CD16+) and plasma levels of six cytokines and LPS among the three study groups. Cocaine users exhibited similar frequencies of monocyte subsets, cytokine levels, and LPS levels compared to controls.

**Conclusions:**

Chronic use of cannabis, but not cocaine, is associated with shifts in non-activated monocyte subset distribution, characterized by increased classical and decreased non-classical monocyte subsets, without concurrent systemic cytokine dysregulation or LPS translocation. These findings highlight substance-specific immune effects, potentially affecting long-term immune function.

## Introduction

Substance use disorders represent a significant global health challenge, contributing to increased morbidity and mortality through both direct and indirect effects on multiple physiological systems, including cardiovascular, nervous, respiratory, and immune systems [1], [2], [3], [4]. Substance use, in general, has been linked to immune dysregulation, characterized by alterations in innate and adaptive immune responses that can exacerbate disease progression and impair host defense mechanisms [5].

Among these substances, cocaine, a widely used illicit central nervous system (CNS) stimulant, demonstrates a complex effect on the immune system. Studies suggest that cocaine exerts direct anti-inflammatory effects on immune cells, such as reducing cytokine production and suppressing myeloid cell activation [6], 7]. However, other studies have reported proinflammatory responses under certain conditions, indicating that cocaine’s immunomodulatory effects may be context-dependent [8], [9], [10]. Overall, the immunomodulatory effects of cocaine contribute to heightened susceptibility to infections and chronic inflammation in chronic cocaine users [11], [12], [13], [14].

Cannabis, which is increasingly used for both medical and recreational purposes, has been reported to exhibit mild to moderate pain relief and anti-inflammatory properties [15], [16], [17]. However, cannabinoid’s immunosuppressive effects have been shown to lead to impaired immune function in long-term users, leading to increased susceptibility to infection [18], 19]. Despite their distinct pharmacological effects and reported immune disruptions, the impact of chronic cocaine and cannabis use on immune cell populations, particularly monocytes and systemic microbial translocation, remains not fully understood.

Monocytes are key players in immune surveillance and inflammation and are classified into three subsets based on surface marker expression: classical (CD14++CD16−), intermediate (CD14++CD16+), and non-classical (CD14+CD16++) monocytes [20]. Each subset performs distinct functions, with non-classical monocytes playing a critical role in vascular surveillance, tissue repair, and mediating proinflammatory responses, especially in chronic inflammatory diseases such as atherosclerosis and neuroinflammation [20]. Systemic microbial translocation contributes to monocyte activation and chronic inflammation [21]. Alterations in monocyte subset distribution and systemic microbial translocation and inflammation have been implicated in various pathological conditions, raising the possibility that chronic cannabis and cocaine use may drive immune perturbations through similar mechanisms.

This study aims to assess the impact of chronic cocaine and cannabis use on monocyte subset distribution and systemic microbial translocation and inflammation, providing insights into substance-specific immune alterations and their potential health consequences.

## Methods

### Study participants and substance use assessment

Participants for this study were recruited from the Medical University of South Carolina (MUSC). The cohort consisted of 5 healthy non-drug-using controls, 11 cannabis users, and 7 individuals with chronic cocaine use, all aged between 18 and 55 years (Table 1). There were differences in age, sex, and race among the three study groups. The MUSC Institutional Review Board approved the study, and all participants provided written informed consent before enrollment. Subjects with a history of or current psychiatric, neurological, or neurodevelopmental disorders, traumatic brain injury, or recent antibiotics or probiotics uses were excluded.

A web-based, self-administered Timeline Followback (TLFB) Method Assessment [22] was used to evaluate participants’ cocaine and cannabis use patterns during the 90 days preceding their study visit. This assessment captured detailed information on the frequency, quantity, and patterns of cocaine and cannabis use, including days of use per month, typical and peak usage periods, routes of administration, age on onset, duration of use, and periods of abstinence.

To confirm self-reported drug use, urine drug screenings were conducted using the onTrak test cup, an *in vitro* diagnostic assay for qualitatively detecting drugs or metabolites in urine. The criteria for chronic substance abuse were followed as described in our previous studies [23], 24], with chronic cannabis use defined as regular use for at least 6 months and chronic cocaine use for at least 3 months. These criteria were verified through self-reported use patterns, TLFB assessments, and urine drug screenings. Chronic cannabis use was identified by meeting DSM-5 criteria for mild to severe cannabis use disorder, identifying cannabis as their main drug via self-report, and a positive urine test for Δ9-tetrahydrocannabinol (THC). Chronic cocaine use was identified by meeting DSM-5 criteria for mild to severe cocaine use disorder and identifying cocaine as their main drug via self-report; a positive urine drug screen for cocaine is not necessary since the half-life of cocaine metabolites is too short. All drug use participants confirmed smoking or snorting but not intravenous administration of cannabis or cocaine.

**Table 1:** Demographic and clinical characteristics of the control and drug users.

	Controls	Cannabis	Cocaine	p-Value
Number	5	11	7	
Sex (male/female)	4/1	9/2	7/0	>0.05
Race (CA/AA)	4/1	8/3	5/2	>0.05
Age (years, mean)	35	40	37	>0.05

CA, Caucasian; AA, African American.

### Flow cytometry analysis of monocyte subpopulations

Peripheral blood mononuclear cells (PBMCs) were isolated from whole blood samples using density gradient centrifugation. Following isolation, PBMCs were stained with a viability marker (LIVE/DEAD^TM^ Fixable Aqua Dead Cell Stain Kit, Invitrogen, Waltham, MA) and monoclonal antibodies targeting CD14 (clone MφP9, BD Biosciences, Milpitas, CA) and CD16 (clone: REA423, Miltenyi Biotec North America, Gaithersburg, MD) to identify monocyte subsets. Monocytes were classified as classical (CD14++CD16−), intermediate (CD14++CD16+), and non-classical (CD14+CD16++). Flow cytometry was performed using a BD Fortessa X20 system, and data were analyzed with FlowJo software (version 10.10.0, BD Biosciences, San Jose, CA).

### Lipopolysaccharide (LPS) and cytokine quantification

Plasma LPS levels were measured using the Pierce^TM^ Chromogenic Endotoxin Quant Kit (Thermo Fisher Scientific, Waltham, MA, USA) according to the manufacturer’s instructions. For cytokine quantification, plasma samples were analyzed for IL-6, TNF-α, IL-10, IFN-γ, IL-2, and IL-4 levels using U-PLEX Biomarker Group 1 (human) Assay kits (Meso Scale Diagnostics [MSD], Rockville, MD). Cytokine measurements were performed following the manufacturer’s protocol, and data were collected using an MSD QuickPlex SQ 120 system.

### Statistical analysis

Data are presented as individual data points with means indicated by horizontal bars. Comparisons among the three groups (non-drug users, cannabis users, and cocaine users) were performed using one-way analysis of variance (ANOVA) followed by post hoc Tukey’s multiple comparisons test to determine statistical significance between groups. A p-value <0.05 was considered statistically significant. All statistical analyses were conducted using GraphPad Prism (version 10.4.1 for macOS, GraphPad Software, Boston, MA, USA, www.graphpad.com).

## Results

### Chronic cannabis users exhibit decreased monocyte activation compared to cocaine users and/or healthy controls, whereas chronic cocaine users exhibit similar monocyte phenotypes compared to controls

**Figure 1:**
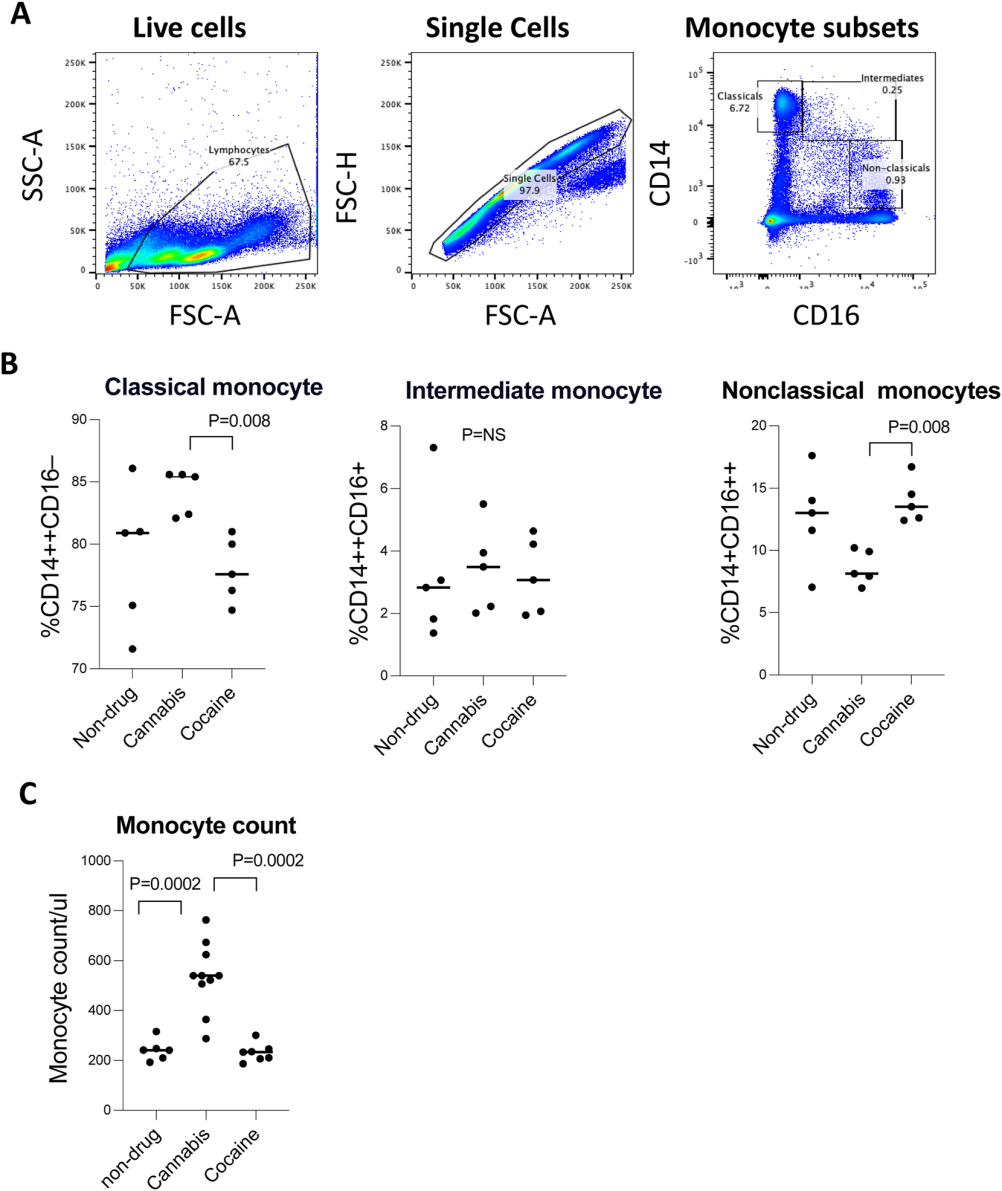
Gating strategy and frequencies of monocyte subpopulation in non-drug controls and chronic drug users. PBMCs from non-drug users, cannabis users, and cocaine users were analyzed via flow cytometry. Our gating strategy (A) included live cell selection (FSC-A vs. SSC-A), singlet discrimination (FSC-A vs. FSC-H), and identification of monocyte subsets based on CD14 and CD16 expression: classical (CD14++CD16−), intermediate (CD14++CD16+), and non-classical (CD14+CD16++). (B) Percentages of monocyte subsets. Classical monocytes were significantly increased, intermediate monocytes showed no significant differences, and non-classical monocytes were significantly decreased in cannabis users compared to cannabis users or controls. (C) Total monocyte counts were significantly higher in cannabis users compared to non-drug users and cocaine users. These differences in monocytes were not determined in cocaine users compared to controls. Data are shown as individual values with medians. One-way ANOVA determined p-values with post hoc Tukey’s test.

To investigate the impact of chronic drug use on monocyte dynamics, we analyzed PBMCs from non-drug users, cannabis users, and cocaine users using flow cytometry. Gating strategies were applied to identify live cells, single cells, and monocyte subsets based on CD14 and CD16 expression (Figure 1A). Monocyte subpopulations were categorized as classical (CD14++CD16−), intermediate (CD14++CD16+), and non-classical (CD14+CD16++).

Our analysis (One-Way ANOVA; p<0.0001) revealed significant overall differences in monocyte subsets across groups. Post-hoc analysis revealed that classical monocytes were significantly increased and non-classical monocytes were significantly decreased in cannabis users relative to cocaine users and non-drug users (p<0.05, Figure 1B). In contrast, intermediate monocytes showed no differences across groups (p=NS). Furthermore, blood total monocyte counts (Figure 1C) were significantly higher in cannabis users compared to both cocaine users and non-drug users (p=0.0002). In contrast, cocaine users did not exhibit significant differences in total monocyte counts relative to controls. These findings suggest that cannabis use is associated with a shift of non-activated monocytes, reflected by increased total and classical monocytes but decreased non-classical monocytes. Cocaine use exhibited similar monocyte subset distribution and total monocyte counts compared to non-drug controls.

### Similar levels are determined in plasma levels of proinflammatory cytokines and LPS between cannabis use and cocaine use

**Figure 2:**
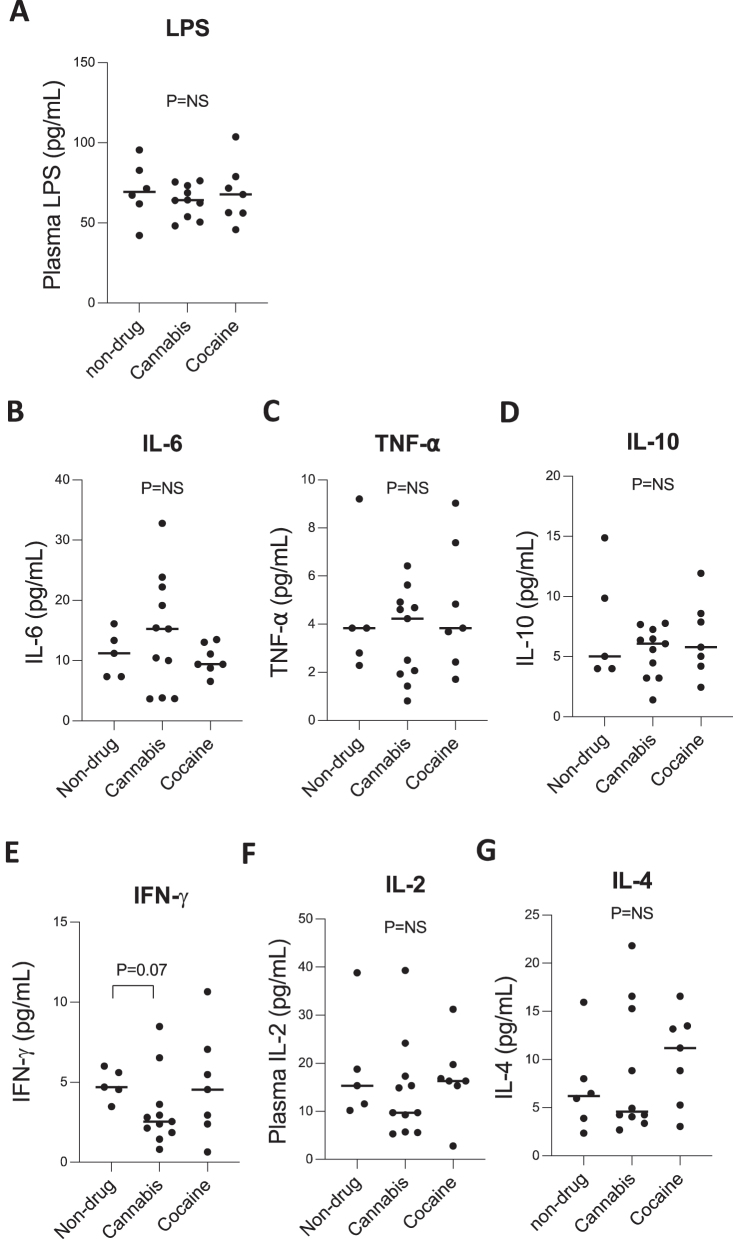
Similar plasma levels of cytokines and bacterial LPS. Plasma samples from non-drug users, cannabis users, and cocaine users were analyzed for (A) plasma lipopolysaccharide (LPS) levels and (B–G) pro- and anti-inflammatory cytokines, including IL-6, TNF-α, IL-10, IFN-γ, IL-2, and IL-4. No significant differences were observed in LPS levels or cytokine concentrations across groups (p=NS for all comparisons), except for a trend in decreased IFN-γ levels in cannabis users compared to non-drug users (p=0.07). Data are shown as individual values with medians. One-way ANOVA determined p-values with post hoc Tukey’s test.

LPS has been linked to monocyte subset changes *in vivo* [21]. To assess whether systemic inflammatory responses and microbial translocation accompanied changes in monocyte subpopulations, we measured plasma levels of LPS and pro- and anti-inflammatory cytokines, including IL-6, TNF-α, IL-10, IFN-γ, IL-2, and IL-4 (Figure 2A–G). There were no differences in plasma LPS levels among non-drug users, cannabis users, and cocaine users (p=NS; Figure 2A). Similarly, systemic cytokine levels were similar across groups for IL-6 (Figure 2B), TNF-α (Figure 2C), IL-10 (Figure 2D), IL-2 (Figure 2F), and IL-4 (Figure 2G) (p=NS for all comparisons). A trend toward reduced IFN-γ levels was observed in cannabis users compared to non-drug users (p=0.07; Figure 2E), though this did not reach statistical significance.

Our study indicates that despite alterations in monocyte subpopulations in chronic cannabis users, chronic cocaine and cannabis use exhibit no changes in systemic inflammatory cytokines or LPS.

## Discussion

Cocaine use is linked to increased susceptibility to infections and chronic inflammatory conditions, while cannabis use often exhibits anti-inflammatory effects [25], [26], [27]. Yet, the mechanisms driving these immune alterations remain unclear. Our findings reveal substance-specific monocyte alterations, with cannabis users exhibiting elevated total monocyte counts and classical monocytes. In contrast, no changes in monocyte phenotypes were observed in cocaine users. All occur without changes in plasma LPS and systemic cytokine levels among the three study groups.

The current study reveals chronic cannabis use-associated monocyte subset shifts occurred without corresponding changes in systemic microbial translocation and cytokines (IL-6, TNF-α, IL-10, IFN-γ, IL-2, and IL-4, Figure 2A–G). The absence of systemic inflammation elevation and monocyte activation contrasts with specific models of cocaine-associated neuroinflammation but may reflect localized immune activation not captured in peripheral blood.

Non-classical and intermediate monocytes play critical roles in vascular surveillance, tissue repair, and pro-inflammatory responses. Their expansion is linked to a heightened risk of chronic inflammatory conditions, including cardiovascular diseases and neuroinflammation [2], 12], 20], 28]. We observed elevated total monocyte counts and classical monocytes, as well as reduced frequencies of non-classical monocytes in cannabis users, a finding consistent with a role of cannabis in anti-inflammatory effects in previous studies [29], 30]. Nonetheless, the increase in classical monocytes and total monocyte counts may reflect immune dysregulation associated with chronic cannabis use. Clinically, a considerable increase in monocytes, also known as monocytosis, is often associated with autoimmune diseases and increased susceptibility to infections, especially in those with immunocompromised conditions like HIV infection [19], 31], 32]. However, frequencies of intermediate monocytes were shown to increase in the blood of cannabis users in a previous study [30], which was not determined in our study. Further research is needed to determine whether this shift in monocyte distribution contributes to protective or suppressed immune responses in cannabis users.

Potential mechanisms underlying these differential effects may include receptor-mediated associated with specific drug use. Regarding cannabis use, cannabinoid receptor-mediated signaling modulates immune cell differentiation and cytokine production, where cannabinoids, such as THC and cannabidiol (CBD), can act on CB2 receptors expressed on immune cells [33]. These interactions lead to complex immunomodulatory effects that alter monocyte distribution, survival, and function, potentially increasing classical and total monocyte counts [34].

In contrast, cocaine’s effects involve chronic sympathetic nervous system activation, leading to elevated catecholamine levels (i.e., norepinephrine) and β2-adrenergic receptor stimulation on monocytes [35]. Non-classical monocytes are shown to express higher levels of β2-adrenergic receptors than classical monocytes, we hypothesize that indicating that chronic cocaine use could may mediate an increase in this monocyte subpopulation locally (i.e., brain, gut) but not reflect in the circulation levels [36]. Ultimately, our findings suggest a mechanistic distinction in the immune-modulating effects of cannabis and cocaine, contributing to the differences in monocyte subset shifts.

Our previous study revealed that chronic cocaine use was associated with the enrichment of Gram-positive bacteria [37]; thus, similar translocated levels of LPS in cocaine users and controls found in the current study do not exclude microbial translocation as a contributing factor to local monocyte or macrophage activation in cocaine users. Other microbial components, such as peptidoglycan (PGN), lipoteichoic acid (LTA), or bacterial DNA, may translocate into the bloodstream via a compromised barrier and engage pattern recognition receptors (PRRs) like TLR2 or TLR4 or the cGAS-STING pathway, potentially driving localized inflammation due to concentrated microbial antigens [38], [39], [40]. This possibility is particularly relevant given the known disruption of mucosal barriers in cocaine users, which may facilitate the translocation of microbial products [41]. However, evidence of systemic microbial translocation in chronic cocaine and cannabis use in humans *in vivo* is lacking.

While our findings provide valuable insights into monocyte subset alterations associated with chronic cannabis versus cocaine use, certain limitations should be considered. The cross-sectional design limits causal inferences, and the small sample size may limit the ability to detect subtle immune changes, particularly among monocyte subpopulations and cytokine responses. Future studies with larger, more diverse cohorts must investigate the underlying mechanisms driving these monocyte alterations and their functional implications.

Furthermore, the absence of tissue-specific immune assessments may overlook localized microbial translocation and inflammatory responses that are not evident in peripheral blood. Future studies should investigate the functional implications of altered monocyte subsets, particularly non-classical monocytes, in tissue-specific contexts during chronic cannabis and cocaine use. Additionally, exploring other markers of microbial translocation and conducting longitudinal analyses could further elucidate the mechanisms driving immune dysregulation in chronic cocaine users and cannabis users.

Our findings reveal that chronic cannabis but not cocaine use alters peripheral monocyte subpopulations and a total monocyte count without elevations in systemic cytokines or plasma LPS levels. Cannabis users exhibited increased total and classical monocyte counts and decreased non-classical monocytes. Given the role of classical monocytes in anti-inflammatory resolution, the changes observed in cannabis users may reflect dysregulated immune modulation. Cocaine-mediated inflammation was observed in prior studies [8], 9], 13], 42], 43]. The different results may stem from the following factors: (1) human *in vivo* studies versus animal studies, (2) *in vivo* versus *in vitro* studies, and (3) assessments using local tissue samples versus blood samples, which may dilute the effects in the local site.

Regarding results of chronic cocaine use in the current study, the unchanged monocyte subset distribution and inflammation in the circulation may suggest potential localized immune activation and inflammation via cocaine-induced disruption of barriers, possibly mediated by non-classical monocytes or other myeloid cells (i.e., microglia), contributing to the heightened risk of cardiovascular and chronic inflammatory conditions in chronic cocaine users [1], 44]. While systemic immune activation markers were unchanged, tissue-specific immune dysregulation or localized inflammation may still occur. Other microbial components, such as PGN, LTA, or bacterial DNA, may contribute to monocyte activation and local inflammation through pathways not captured in this study. Future research should investigate the functional roles of altered monocyte subsets, microbial translocation beyond LPS, and tissue-specific immune responses to understand cocaine-associated immune dysregulation better compared to cannabis.

## Supplementary Material

Supplementary Material Details
